# Global health impacts of policies: lessons from the UK

**DOI:** 10.1186/1744-8603-10-13

**Published:** 2014-03-10

**Authors:** Modi K Mwatsama, Sidney Wong, Dena Ettehad, Nicola F Watt

**Affiliations:** 1 UK Health Forum, London, UK; 2 NHS, Public Health Specialist, London, UK; 3 King’s College London, School of Medicine and Dentistry, London, UK; 4 European Centre on Health of Societies in Transition, London School of Hygiene and Tropical Medicine, 15-17 Tavistock Place, London WC1H 9SH, UK

**Keywords:** Global health, Impact assessment, Policy coherence, Development

## Abstract

**Background:**

The UK government committed to undertaking impact assessments of its policies on the health of populations in low and middle-income countries in its cross-government strategy “Health is Global”. To facilitate this process, the Department of Health, in collaboration with the National Heart Forum, initiated a project to pilot the use of a global health impact assessment guidance framework and toolkit for policy-makers. This paper aims to stimulate debate about the desirability and feasibility of global health impact assessments by describing and drawing lessons from the first stage of the project.

**Discussion:**

Despite the attraction of being able to assess and address potential global health impacts of policies, there is a dearth of existing information and experience. A literature review was followed by discussions with policy-makers and an online survey about potential barriers, preferred support mechanisms and potential policies on which to pilot the toolkit. Although policy-makers were willing to engage in hypothetical discussions about the methodology, difficulties in identifying potential pilots suggest a wider problem in encouraging take up without legislative imperatives. This is reinforced by the findings of the survey that barriers to uptake included lack of time, resources and expertise. We identified three lessons for future efforts to mainstream global health impact assessments: 1) Identify a lead government department and champion – to some extent, this role was fulfilled by the Department of Health, however, it lacked a high-level cross-government mechanism to support implementation. 2) Ensure adequate resources and consider embedding the goals and principles of global health impact assessments into existing processes to maximise those resources. 3) Develop an effective delivery mechanism involving both state actors, and non-state actors who can ensure a “voice” for constituencies who are affected by government policies and also provide the “demand” for the assessments.

**Summary:**

This paper uses the initial stages of a study on global health impact assessments to pose the wider question of incentives for policy-makers to improve global health. It highlights three lessons for successful development and implementation of global health impact assessments in relation to stewardship, resources, and delivery mechanisms.

## Background

Health Impact Assessments emerged as the recommended tool for maximising the health of the population through embedding health in all policies with the publication of the Gothenburg consensus. The framework, which was produced by the World Health Organization [WHO] European Centre for Health Policy, was underpinned by four core values: sustainable development, equity, democracy and the ethical use of evidence [1]. The United Nations Special Rapporteur on the Right to Health has also recommended that countries integrate human rights impact assessments within the policy making process in order to support the realisation of the right to health [2-4].

The field of Health Impact Assessment of policies within domestic or national borders has largely developed in the industrialised world, supported in Europe through policies such as the “Health in All Policies” theme of the Finnish Presidency of the European Union in 2006 and the European Commission health strategy 2008–13 [1]. By contrast, a comprehensive review of the literature has demonstrated that the field of global or international health impact assessments has remained relatively under-developed [5]. Among the key actions required to address the root causes of global inequalities, the WHO Commission on Social Determinants of Health called on countries to institutionalise health equity impact assessments and mitigation as part of the development of national level policies [1,4,6]. One recent report determined that in principle, existing health impact assessment methodology should be sufficient for this purpose [7]. However, few countries assess the global cross-border impacts of their own policies and within developing countries, national health impact assessment capacity development has been largely neglected.

Within the European Union [EU] context, there have been relevant discussions about the need to ensure that policies adopted at the EU level support, or at a minimum do not conflict with, development goals [8]. Indeed Article 208 of the Lisbon Treaty states: “The Union shall take into account the objectives of development cooperation in the policies that it implements which are likely to affect developing countries”. The EU established the principle of Policy Coherence for Development in recognition that “some of its policies can have a significant impact outside of the EU and that either contributes to or undermines its development policy.” As part of this process, the EU “seeks to minimise contradictions and to build synergies between policies other than development cooperation that have an impact on developing countries, for the benefit of overseas development”. [9] The EU has also adopted the use of dedicated trade Sustainability Impact Assessments to assess the social, environmental and economic impacts of trade liberalisation on areas such as health and education [10]. However there is no specific instrument or toolkit identified to facilitate impact assessments of EU policies on international development more broadly across departments within the European Commission. Both the principle of Policy Coherence for Development and Sustainability Impact Assessment tools are aligned with the objectives of global health impact assessments in that they all focus on considering the global, cross-border impacts of EU or UK policies and actions on developing countries – albeit on different sectors or priorities.

At the global level, while global health impact scrutiny of international policies and structures such as the International Monetary Fund [4] remains under-utilised, consensus is emerging among the United Nations [UN] institutions around the need for policy coherence for development. In a report to the UN Secretary General on the post 2015 development agenda, the UN system-wide Task Team recommended that “a high degree of policy coherence would be required at the global, regional, national and sub-national levels” to support the achievement of equitable and sustainable development for all [11].

The UK government is the first to explore the use of impact assessments to take greater account of the global health impact of foreign and domestic policies. The purpose of a global health impact assessment is to assess the impact on health and social determinants of health of a particular country’s policies on populations in low and middle-income countries. These complex global impacts are often unconsidered, and/or forgotten owing to their complexity, issues to do with available evidence, information and data, as well as lack of an awareness of global impacts among national policy-makers. Through the implementation of the UK cross government strategy, Health is Global [12] (since updated through the publication of a corresponding outcomes Framework [13]), the Department of Health, in collaboration with the National Heart Forum (now UK Health Forum), initiated a project to develop and support the use of global health impact assessments (GHIA). With the aim of stimulating discussion and debate about the desirability and feasibility of such assessments, this paper reports on the learning from the first stages of this project, developing a guidance framework for policy-makers and identifying the perceived barriers to implementation.

## Discussion

### The context – developing a UK framework for global health impact assessments

#### Purpose and functions of global health impact assessments

An initial literature review was conducted to identify whether GHIA tools already existed, and inform the development of such a tool. The review included searches on scientific databases including Medline and Web of Science using key words including international health, global health, development and impact assessment and reviewing the references and bibliographies of articles identified. An online survey was also circulated to academic health impact assessment networks through existing contacts and the Health Impact Assessment net, http://www.jiscmail.ac.uk to identify relevant institutions, websites and grey literature resources.

The literature review showed that whilst there are many instances of international bodies, such as the World Bank’s International Finance Corporation, carrying out health impact assessments of specific development projects relevant to a particular country or community situation [1], there are no existing published methodologies or toolkits for a GHIA, i.e. for understanding the impact of a stakeholder’s or government’s mainstream policies on global health, or the health situation across borders. In light of the paucity of published literature in this field, and to mitigate potential resistance of policy officials to using unfamiliar methods, the methodology and guidance for GHIA were therefore based upon and complemented the Department of Health domestic health impact assessment toolkit [14]. This toolkit was published following a review of how HIA was carried out across the UK Government [15] so although no evaluation of the toolkit itself has been identified, it was designed on the basis of evidence about UK policy-making. The GHIA tool used the same five basic steps included in the domestic HIA tool which are considered best practice in the HIA literature: screening, identifying health impacts, prioritising the impacts, quantifying those impacts and recommendations for policy-makers.

Several conclusions from this initial review informed the design of the tool. Firstly, although there is no theoretical limit to the geographical scope of a GHIA, the priority would be to focus on low and middle income countries, which tend to be both disproportionately affected by negative impacts from other countries’ policies, and less able to mitigate against such impacts, as exemplified in the case of climate change [16]. A GHIA should be a decision support tool, guiding policy-makers to consider the positive and negative impact of their proposed policy on the determinants of health, people’s individual ability to improve their health and well-being, and people’s access to health and social care. It should identify any unintended health consequences that may either lend support to the policy in question or suggest improvements to it. Finally, it should also contain a clear analysis of the segments of the population that will be most affected, in order to ensure appropriate regard for equity considerations.

### The global health impact assessment tool

The GHIA tool was developed to be applicable to a wide variety of policy such as climate change, trade, food security, illegal drugs, health worker migration, access to medicines and conflict impacts on health. A brief example of the application of the tool to Agriculture and Climate Change Policy is given in Table 1. As demonstrated by the climate change example, the global health impacts of domestic policies often overlap, and are inter-related. Achieving UK climate change mitigation targets will require efforts in the UK transport and agriculture sectors among others. The resulting health benefits from action will not just be limited to low and middle income countries, but benefits will also accrue domestically in terms of health improvements, for example, from reductions in non-communicable diseases.

**Table 1:** Example framework for a global health impact assessment of a hypothetical policy relevant to climate change

**Background**	**Global impacts of climate change, transport, and agriculture policy on health**
	Greenhouse gasses [GHG] are a major determinant of global warming and climate change, with temperatures forecast to rise by at least two degrees Celsius by 2050 [17]. The UK government’s Carbon Plan outlined efforts towards the achievement of an 80% reduction in UK GHG by 2050 through a number of sectors including agriculture, transport, low carbon buildings [heating and energy efficiency], and low carbon industry [manufacturing and steel] [18]. While the plan modelled economic impacts of climate change, it did not extend these to include the “social externalities” such as health impacts in the UK or globally [13]. Among the adverse effects of climate change, increased droughts, floods, storms and heat waves are forecast to pose a serious challenge to human well-being, health and equity [19,20]. Relevant policies might include agriculture and transport: in the UK domestic transport accounts for around 21% of GHG emissions, while agriculture accounts for 9% of emissions [21]. Worldwide, the agriculture sector is estimated to account for 10-12% of total GHG emissions [22], while agriculturally induced change in land-use such as deforestation and conversion of pasture to arable land for feed production accounts for a further 6-17% of GHG [22].
**STAGE 1**	**Screening:** in the identified countries, will the policy affect a] access to health and social care, b] people’s individual ability to improve their own health and wellbeing or c] the wider determinants of health?
	Approximately 150,000 deaths and 5.5 million Disability Adjusted Life Years worldwide were attributed to climate change in 2000 [23]. Over 85% of these adverse health impacts occurred in developed countries [23], disproportionally affecting the socially disadvantaged including elderly, children, coastal populations and urban slum dwellers [19]. Unless serious efforts are taken to tackle climate change these impacts will rise drastically and serve to widen inequalities. Therefore where a policy is thought likely to have significant positive or negative effects on climate change, this would trigger the need for a more comprehensive understanding of the health effects of that policy at the screening stage of a GHIA.
**STAGE 2**	**Identify health impacts:** establish causal links; use WHO, World Bank and Department for International Development resources; consult policy experts and literature
	The likely impacts of climate change on health include [24]:
	● Increased deaths and disease events, including cardiovascular events and heat-stroke from temperature extremes – including very hot and very cold days
	● Increased allergic disorders such as hay-fever and asthma, due to longer pollen season
	● Increased water-borne infections such as cholera
	● Increased food poisoning and diarrhoeal disease, eg from salmonella, due to higher temperature
	● Increased vector-borne diseases from mosquitoes and ticks
	● Population displacement and lost livelihoods due to sea-level rise
	● A rise in conflict and instability due to resource scarcity, including food and water
	Such impacts should therefore be considered in the context of the proposed policy, according to the degree to which the policy is expected to have a global impact.
**STAGE 3**	**Prioritise important health impacts,** including consideration of differential and cumulative impacts on populations
	The health impacts identified at stage two that are relevant to the proposed policy should then be prioritised according to their likelihood and magnitude.
**STAGE 4**	**Quantify health impacts (analysis):** describe, quantify and/or monetise priority health impacts
	Example benchmark statistics that might be relevant in such a GHIA:
	Infectious diseases: By 2030, diarrhoeal diseases are forecast to increase by 10% primarily in young children – from a 2000 baseline – owing to climate change [25], while seasonal exposure to malaria is expected to increase by 16% to 28% in Africa by 2100 [25]. The costs of treating rotavirus diarrhoea in India are in the region of $41–72 million a year [26], while Malaria is estimated to cost Africa about $12 billion per year in lost gross domestic product [27].
	Food security: In some developing countries, agriculture yields are forecast to reduce by up to 50% as a result of climate change, with significant impacts on food security, hunger and nutritional well-being, especially among the poor [16,19].
	Insecurity and civil unrest: As climate change leads to rising costs of fuel, water and food, the risk of social unrest and security will be aggravated [19], particularly in conflict affected regions [8].
**STAGE 5**	**Recommendations to improve policy:** including remediation, suggested policy changes and ways of monitoring the on-going health impact to promote accountability and ownership.
	According to the policy under consideration and the priority health impacts identified, relevant recommendations might include e.g. technological changes, strategies to modify agricultural activity or transport usage, or wider policy changes such as influencing Common Agricultural Policy reform.

### Facilitators and barriers to implementing global health impact assessments

In developing methods for capacity building, we took account of a review of domestic health impact assessments carried out by various government departments [15]. This primarily highlighted the types of support needed for policy-makers undertaking health impact assessments, for example the need for more training and increased access to advice. A survey was carried out of policy-makers (which included policy leads and members of their teams) from a range of relevant policy areas within government departments in order to assess previous experience of impact assessments, preferred support mechanisms for undertaking GHIAs, and the main challenges and barriers they would envisage. The survey was disseminated by email to policy-makers in government and academics who had worked with policy-makers on health impact assessments. The survey was directly emailed to the 73 participants who had attended the launch event for the Department of Health report *“Putting health in the policy picture: Review of how health impact assessment is carried out by government departments”*[15]; it was emailed to Impact Assessment leads within each government department for dissemination to policy-makers in their departments; and it was emailed to the 20 members of a strategic cross-government global health network convened by the Department of Health. Twenty responses were received to the survey. While the number was small, the responses provided insight into the perspectives of those policy-makers who were particularly interested in global health impact assessments. 15 out of 18 survey respondents had undertaken an impact assessment in the past, of which equality impact assessments, followed by health impact assessments were the most common.

In terms of facilitators, in line with the domestic HIA review, supporting guidance and documents were identified as the most useful tools to support impact assessments (15/15 respondents), followed by web-based resources. Experts and training were also cited as being useful. These findings were in line with discussions with policy-makers. All policy-makers and teams surveyed were encouraged to pilot the GHIA guidance framework and toolkit, and personal contacts in government were also approached. While no additional financial resources were made available, support in the form of dedicated staff time was offered to support the early adopters.

Findings from the survey were triangulated with themes which emerged from discussions with policy-makers as part of the process of identifying volunteers to pilot the tool. Potential participants for discussion were identified through the policymaker survey described previously. In addition, government websites were reviewed for open or forthcoming consultations which were obviously amenable to GHIA and a list of seven potential policies was compiled. The policy leads for the consultations were then contacted for a discussion. Snowball sampling was used to identify further individuals or teams working on policies which would lend themselves to global health impact assessments. A total of 8 discussions took place involving a mix of face-to-face or telephone meetings with up to three members of the policy teams (at their offices) and one or two of the authors over a period of three months. A group discussion was held with the members of the strategic cross-government global health network during a dedicated session on global health impact assessments. The discussions were summarised and thematically analysed.

The project was unsuccessful in identifying a policy team to pilot the toolkit and at the time of writing the project is effectively suspended. The online survey and subsequent discussions with policy-makers identified a number of challenges and barriers to the use of GHIAs at policy level:

•GHIAs are not a mandatory process

•Policy-makers do not realise the value of a GHIA

•Lack of staff and resources to dedicate to undertake them

•Wrong stage of the policy development cycle

•In some cases, political sensitivities relating to the policy under development meant that a GHIA would be inappropriate

•Lack of expertise and knowledge of health impact assessment

•Lack of time and competing work priorities.

### Three key lessons for institutionalising global health impact assessments

In line with stated UK Government global health policy, our initial literature searches underlined that there is a genuine gap for GHIA to assess the impacts of policies on less developed countries, particularly for the most vulnerable. The analysis of national policies in terms of their global health impacts is therefore a valuable, yet under researched field. Our investigations found no evidence of existing practice or theory. Whilst domestic health impact assessments have some leverage through their incorporation within the mandatory impact assessments, substantial barriers remain to their implementation, finalisation and testing as set out above.

From our experience, it appears that a global health impact assessment as originally envisaged as a separate test, aligned to the greatest extent possible with the domestic health impact assessment, is currently untenable within the UK context. The existence of a tool and guidance alone will not be enough to ensure assessments are carried out. Wismar et al. [28,29] use a health systems model to analyse the elements needed to support institutionalisation: governance or stewardship, funding and financing, resource generation and delivery. These align well with the findings of a study by CONCORD - The European NGO confederation for relief and development [8], in terms of what was needed for Policy Coherence in Development to happen. Together with our own experience, these therefore lead us to three key recommendations for the future development and implementation of global health impact assessments.

#### 1. Identify a lead government department and champion

Successful institutionalisation of global health impact assessments will depend on government buy-in as highlighted by CONCORD who note that there is a need for “Continued political will to translate into the right policy choices favourable to poor people in developing countries”. Within the EU, a Standing Rapporteur on Policy Coherence for Development was appointed to incorporate these principles within the parliamentary process [8]. Arguably, the Department of Health has been willing to show leadership on this issue by its pursuit of this work stream. However, securing cross-government buy-in and uptake has proved more challenging. A high-level cross-government leadership mechanism, such as the former UK Cabinet subcommittee on public health (disbanded in 2012), could potentially be a useful mechanism for furthering such an agenda. If the experience of developing the UK’s overall global health strategy is anything to go by, political leadership from the highest level may well be needed [30].

Mandating GHIAs across government at the national level would be an attractive option in the long term, to ensure uptake. That said, although the downside of not being mandatory is that it fails to encourage maximum implementation, it does allow flexibility, which is critical given the need for further methodological development in this area.

#### 2. Obtain adequate resources and maximise their potential, including through integration

These two factors are considered together here, since for the foreseeable future, support for implementation will have to come within existing budgets. The need for appropriate resourcing was also identified in the CONCORD study, described as the “capacity to carry out the assessments, supported by education, appropriate tools etc.” [8].

It is in this area that there is the most potential to look for efficiencies and incentives. Most government departments will have some capacity for impact assessments, but none will have budgeted for the additional demands of non-mandatory tests. Integrating GHIAs into other processes would be an obvious step to increase the appeal of the tool to policy-makers. Indeed, there is already discussion in the literature about the pros and cons of the domestic health impact assessments being, as is currently the case in the UK, free-standing, or whether to integrate them within the strategic environmental assessment [31-34]. There are clearly advantages and disadvantages to each. In the UK context, the main advantage of integration would be the additional pressure of mandating the test, the main disadvantage being inflexibility and a risk that health might be marginalised. Although draft guidance on integration was consulted on and positively reviewed [33], no finalised Department of Health guidance has been published. On balance, though, it is clear that there are substantial barriers to implementing and institutionalising a free-standing tool [29], and these are considerably magnified in the global health impact assessment context as we found.

Global impact assessment advocates should investigate ways to embed the goals and principles of GHIAs into existing processes, in particular, where there is an existing legal and/or policy lever, for example the Integrated Impact Assessment or Strategic Environment Assessment. Attention should also be paid to developing appropriate skills, within and outside the Department of Health, for carrying out and engaging with such assessments.

#### 3. Develop an effective delivery system involving both state and non-state actors

Wismar’s analysis consists of four elements - a lead agency, someone to actually carry out the assessment, a link between the ownership of the decision and the trigger for process, and a link between assessment and reporting. Since global health impact assessment is still very much in its infancy, it is likely that those who are involved form only a very small group and therefore these linkages and clarity of roles should be automatic. However, to this list we will add the need for involvement of civil society actors including non-governmental organisations (NGOs) – as indicated in the CONCORD study. Advocacy – and scrutiny – will be necessary to ensure a “voice” for the constituencies who may be otherwise affected by government policies and to provide the “demand” for assessments. While examples relating to global health are scarce, development NGOs such as Oxfam have successfully advocated on behalf of affected constituencies in the area of trade and development policy. Development NGOs including Oxfam were instrumental in bringing about reform of the European Common Agriculture Policy sugar regime in 2006, which led to increased access by sugar producers from selected low income countries to the European market, following decades of unfair subsidies and tariffs favouring European sugarbeet producers [35,36]. Given that further methodological development is needed, and that the familiarity of policy-makers with appropriate sources of information about global impact may be limited, the expertise of other partners will be crucial. This should include all those with experience and evidence about the potential for improving and protecting public health worldwide, including in low and middle income countries: this would therefore encompass, in our view, civil society actors including NGOs, academics and global health and development experts.

## Summary

Global health impact assessments aim to assess the impact on health and social determinants of health of a particular country’s policies on populations in low and middle-income countries. The UK government committed to undertaking GHIAs on the health of populations in low and middle-income countries in its cross-governmental strategy “Health is Global.” To facilitate this process the Department of Health in collaboration with the National Heart Forum, initiated a project to pilot the use of GHIAs. A literature review was followed by discussions with policy-makers and an online survey about potential barriers, preferred support mechanisms and potential policies on which to pilot a global health impact assessment toolkit. The project recognised that GHIAs share many of the same barriers to implementation as domestic health impact assessments, including failure to appreciate their value, limited expertise, lack of time and resources and competing priorities, which were compounded by GHIAs not being mandatory.

Analysis to date of barriers and facilitators leads to three initial recommendations for GHIA development and implementation:

1. Identify a lead government department and champion – to promote uptake of GHIAs across government, and champion their value.

2. Obtain adequate resources and maximise their potential.

3. Develop an effective delivery system involving both state and non-state actors – civil society non-governmental organisations will be particularly key to represent the global ‘voices’ of those affected as well as drive external demand for GHIAs.

This work underlines the desirability of continuing to develop and implement GHIA. We welcome debate and look forward to GHIA being higher on the academic and political agenda in the future.

## Competing interests

The authors declare they have no conflicts of interests.

## Authors’ contributions

MM, NW and SW wrote the initial and subsequent drafts of the manuscript. DE commented on and edited drafts of the manuscript. All authors read and approved the final manuscript.
